# Robust Temporal Averaging of Time Intervals Between Action and Sensation

**DOI:** 10.3389/fpsyg.2019.00511

**Published:** 2019-03-19

**Authors:** Huanke Zeng, Lihan Chen

**Affiliations:** School of Psychological and Cognitive Sciences, Beijing Key Laboratory of Behavior and Mental Health, Peking University, Beijing, China

**Keywords:** temporal averaging, action, auditory, visual, interval

## Abstract

Perception of the time interval between one’s own action (a finger tapping) and the associated sensory feedback (a visual flash or an auditory beep) is critical for precise and flexible control of action and behavioral decision. Previous studies have examined temporal averaging for multiple time intervals and its role for perceptual organization and crossmodal integration. In the present study, we extended the temporal averaging from sensory stimuli to the coupling of action and its sensory feedback. We investigated whether and how temporal averaging could be achieved with respect to the multiple intervals in a sequence of action-sensory feedback events, and hence affect the subsequent timing behavior. In unimodal task, participants voluntarily tapped their index finger at a constant pace while receiving auditory feedback (beeps) with varied intervals as well as variances throughout the sequence. In crossmodal task, for a given sequence, each tap was accompanied randomly with either visual flash or auditory beep as sensory feedback. When the sequence was over, observers produced a subsequent tap with either auditory or visual stimulus, which enclose a probe interval. In both tasks, participants were required to make a two alternative forced choice (2AFC), to indicate whether the target interval is shorter or longer than the mean interval between taps and their associated sensory events in the preceding sequence. In both scenarios, participants’ judgments of the probe interval suggested that they had internalized the mean interval associated with specific bindings of action and sensation, showing a robust temporal averaging process for the interval between action and sensation.

## Introduction

Time perception upon the interval between one’s action and its sensory feedback (such as visual flash or auditory beep), i.e., sensorimotor timing, is critical for daily perception, behavioral decision and even human living (Repp, 2005). Two prominent examples of sensorimotor timing are sensorimotor synchronization (Aschersleben and Bertelson, 2003; Repp, 2005, 2006a,b) and temporal recalibration effect (TRE) (Stekelenburg et al., 2011; Sugano et al., 2012, 2014, 2016, 2017). In sensorimotor synchronization, observers produced tapping movements in synchrony with a sequence of isochronously (and continuously) repeated pacing signals, being either light flashes or auditory beeps (Aschersleben and Bertelson, 2003). A typical finding in sensorimotor synchronization is that timing of the taps has been biased significantly to the auditory signals than visual flashes, when the taps were synchronized with continuous visual or auditory stimuli, indicating the preference of the perceptual system for continuous information with visual stimuli (Varlet et al., 2012; Armstrong and Issartel, 2014). TRE, on the other hand, reflects the nature of “causality” between action and its sensory feedback, and time adaptation aftereffect. In a seminal study, Stetson et al. (2006) inserted a temporal delay between one’s own action (key presses) and the associated sensory feedback (visual flashes). Following a period of adaptation, when the flashes appeared unexpectedly after the keypresses, however, they were often perceived as occurring before the keypresses (Stetson et al., 2006), demonstrating recalibration effect for motor-sensory temporal order judgments.

In a typical sensorimotor synchronization task, observers are usually tapping according to the pacing signals with regular rhythm. However, it is often the case that the pacing rhythm is not regular, wherein observers have to calculate the “mean” rhythm (as a temporal reference) for making the subsequent prompted action decision and execution, whether by adopting the temporal estimation or (re)production tasks. The ability to extract the average time interval information in the action-sensory feedback sequence demonstrates the individual timing sensitivity (“temporal window” for sensory integration) and help us adapt to the environmental changes (Repp, 2005). The computation of the “mean,” i.e., temporal averaging process, has been realized in a number of contexts, including crossmodal interaction in recent studies (Cheng et al., 1996; Matell and Henning, 2013; Schweickert et al., 2014; De Corte and Matell, 2016a; Chen et al., 2018). One compelling example for temporal averaging is the central tendency effect within the broader framework of Bayesian optimization. In the central tendency effect, observers incorporated the mean of the statistical distribution for sensory properties to assimilate/bias the estimates toward the mean (Jazayeri and Shadlen, 2010; Burr et al., 2013; Shi et al., 2013; Karaminis et al., 2016; Roach et al., 2017). For examples, the discrimination of the target sensory interval was biased to the preceding time interval from a different modality (Burr et al., 2013), the discrimination of visual apparent motion was modulated by the perceived mean inter-interval in the preceding auditory sequence (Chen et al., 2018; Wan and Chen, 2018).

The perception of the time interval between an action and its sensory feedback, in which the perception of time will be biased to the concurrent actions, is different to the perception of time intervals within pure sensory events. A recent study showed that motor timing during rhythmic tapping influences the visual timing. Tomassini et al. (2018) asked participants to tap their finger with a rhythm same to the preceding sequence of four auditory tones. During finger tapping, they were presented with an empty visual interval and judged its time interval compared with the previously established (internalized) interval of 150 ms. The perceived time was maximally expanded at halftime between two consecutive finger taps and the maximal expansion has been found to be anchored to the center of the inter-tap interval. This distortion in time perception indeed indicates that a timing mechanism exists to maximally keep perception and action accurately synchronized (Tomassini et al., 2018). In another seminal study, Yon et al. (2017) investigated the influence of movement duration on the perceived duration of an auditory tone. The judgments of tone duration were attracted toward the duration of executed movement-the tones were perceived to last longer when participants executed a movement with longer duration (Yon et al., 2017).

Temporal averaging entails the empirical inquiries with regards to the distribution of irregular (unequal) time intervals (De Corte and Matell, 2016a; Chen et al., 2018; Wan and Chen, 2018), selective averaging one of the sequences (Overduin et al., 2008), as well as potential capacity limits of simultaneous temporal processing (Cheng et al., 2014). Schweickert et al. (2014) demonstrated that observers estimated the average of tone durations and their performance was influenced by the distribution of the tone durations. In general the estimated averages were a linear function of the stimulus means. The estimates were accurate for the smallest population mean but underestimates for the larger means, and human observers subjectively shortened the durations in memory (Schweickert et al., 2014). With multiple intervals, human observers could encode two different, and distinct, standard durations. In this case, temporal generalization with respective to the one of the two standards was subject to the memory loading in temporal references as well as their variances (Jones and Wearden, 2004). Moreover, take two consecutively presented standards (A and B, each presented three times, but the duration of B was 100 ms longer than A) for example, the certain combinations of delay and interference could render the memory of A unusable and a new standard (“false memory”) is constructed on the basis of the remembered relationship between A and B (Ogden et al., 2008). Therefore, the internal representation of temporal statistics depends on the distribution of time intervals, the variances of the intervals and is affected by the potentially memory mixing effect (due to the time delay as well as the interference among the many intervals being encoded).

In current study, we examine the mechanisms of temporal averaging of the time intervals between action and its sensory feedback (visual flash and auditory beep). Specifically, we investigated how the mean and irregularity (variances) in the distribution of time intervals affect the perception of target interval in the loop of action and its sensory feedback. Secondly, we examined how human observers can selectively average the sensory-specific time intervals in two sequences in which the actions were bound with either visual flashes or auditory beeps (Chen and Vroomen, 2013). Lastly, we examined the potential memory mixing effect induced by the memory load (and decay) and inherent individual capacity limit of simultaneous temporal processing.

We implemented four experiments to address these issues. In Experiment 1, we examined the ability of extracting the mean interval from a sound sequence and replicated the central tendency effect. In Experiments 2 and 3, we studied the selective temporal averaging in which the actions were bound with two types of events: beeps of two types of pitches, or two types of sensory stimuli (visual flashes and auditory beeps). In Experiment 2, we investigated whether observers could selectively separate the different mean action-auditory feedback intervals and hence make the comparisons between the produced interval and the preceding duration-specific mean auditory intervals. To examine whether the ability of temporal averaging is dependent on the individual modalities (events) or not, in Experiment 3, we used both auditory beeps and visual flashes as sensory feedbacks and examined the selectivity of temporal assimilations to either short or long mean intervals (actions associated with visual or auditory feedbacks). By averaging, human observers could take both the mean interval information and the variance of the intervals into account (Acerbi et al., 2012). In Experiment 4, we further looked into whether the variations of the intervals (by manipulating the coefficient of variances, CV) affect the averaging process of temporal information. The results from the four experiments largely support a robust temporal averaging process for time intervals between actions and their associated sensations. We further validated the effectiveness of the temporal averaging of the intervals rather than the sampling from individual intervals (including the last interval of the action-sensation loops), and discussed the limited role of the memory load on the averaging process with the current paradigms.

## Materials and Methods

### Stimuli and Apparatus

Auditory stimuli in a sound sequence were pure tones (30 ms, 500 Hz or 1000 Hz), with 65 dB SPL. Two pure tones of 2000 Hz were used as cueing signals. The starting cue (duration of 500 ms) prompted the beginning of a trial. The testing cue (for the last tap, duration of 200 ms) indicated the coming of the probe interval for discrimination (see the following procedure for more details).

Visual flash was a black disk (duration of 30 ms, 2.74 degree in diameter, 11 cd/m^2^ in luminance) appearing at the center of the screen, with a gray background (16.8 cd/m^2^ in luminance), presented on a 27-inch screen (ASUS PG278QR, NIVIDIA GeForce GTX 1080 Ti visual graphic card). The viewing distance from the participants to the center of the monitor was 60 cm. Auditory stimuli were delivered through NIVIDIA High Definition Audio. Participants wore headset of Sennheiser Momentum 2 to receive the sounds. We used RTBox v6 (Suzhou Litong Company Limited, China) to collect responses. The experimental program was written with Matlab (Mathworks Inc.) and the Psychophysics Toolbox (Brainard, 1997; Pelli, 1997; Kleiner et al., 2007).

In Experiment 1, only 500-Hz tones were used and mean of eight intervals between tappings and tones (sensory feedback) was 800 ms. The eight sequential intervals were in the time range of 600 to 1000 ms, and were drawn from a Gaussian distribution of N(800, 100). Using customized codes, we composed each trial(sequence) to ensure the coefficient of variance (CV, i.e., the ratio of the standard deviation to the mean) of all intervals was between 0.1 and 0.15, thus to largely randomize the temporal information as well as within the human observers’ perceptual expertise to perform the tasks. In Experiment 2, two mean intervals were used. The short interval (mean of 400 ms) was associated with low-pitch tone (500 Hz) and the long interval (mean of 800 ms) was associated with high-pitch tone (1000 Hz). The short sequential intervals were in the range from 200 to 600 ms, and were drawn from a Gaussian distribution of N(400, 100). The CV of the intervals was between 0.1 and 0.15. The mapping between tone pitch and mean interval was reversed in the other condition. In Experiment 3, the similar configurations were used as in Experiment 2 except that both auditory and visual feedbacks were used. In Experiment 4, we designed two types of tap-tone sequences in which the mean tap-tone interval was kept at 800 ms. However, for one sequence, the taps were followed with tones (500 Hz) with low CV (between 0.1 and 0.15) of the intervals. For the other sequence, the taps were associated with tones with high-pitch tones (1000 Hz) and with high CV (between 0.3 and 0.35). The CVs were determined by previous evidence so that in this range human observers could well perform the relevant tasks (Chen et al., 2018; Getty, 1975a,b). For all the above experimental conditions, following the sequences of action-sensory feedback, participants pressed a button and generated an interval of 200, 400, 600, 800, 1000, 1200, or 1400 ms, to compare with the preceding long mean interval (800 ms); and from 100, 200, 300, 400, 500, 600, or 700 ms to compare with the preceding short mean interval (400 ms).

In the formal experiments, the preceding sequence contained two different intermixed durations, with the two different durations each cued by different pitches or by different sensory events (visual flashes or auditory beeps). Under this context, people can extract and maintain a standard for each duration. The two standards might interact and may interfere a bit in memory references. To examine whether there are perceptual shifts and response biases due to the mixing of the two sequences (standards), we further implemented control tests with the same tasks as in formal experiments, but obtained the baseline data for mean 400 and 800 ms interval conditions from another groups of participants.

### Procedure

The experiments were performed in compliance with the institutional guidelines set by the Academic Affairs Committee, School of Psychological and Cognitive Sciences, Peking University. The protocol was approved by the Committee for Protecting Human and Animal Subjects, School of Psychological and Cognitive Sciences, Peking University. All participants gave written informed consent in accordance with the Declaration of Helsinki, and were paid for their time on a basis of 40 CNY/hour, i.e., 6.3 United States dollars/hour.

**FIGURE 1:**
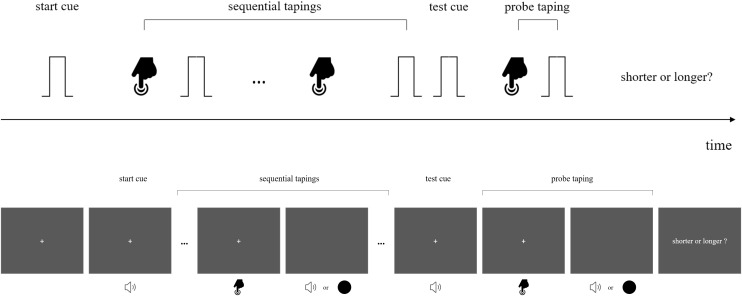
Stimuli configurations and schema for the experiments. (Upper): Experiments 1, 2, and 4. In a typical trial, upon hearing a beep participants voluntarily pressed a button to issue its sensory feedback (“beeps,” with same or different pitches). When the sequence of multiple action-sensory events was over, another signaling beep appeared which prompted the participants to issue a press and it was followed by a last sensory feedback. At this time point they were encouraged to make perceptual discrimination of whether the probe interval (between the offset of the action and onset of the beep) was shorter or longer than the mean interval between the action and its sensory feedback. (Down): The procedure for Experiment 3. The general procedure was the same as shown in the upper figure, however, the sensory feedback include mixed streams of visual flashes and auditory beeps. Participants were asked to compare the probe interval between tap and flash, or between tap and beep with the corresponding mean interval of the preceding intervals of the same type. Detailed information was given in the main text.

In a preceding action-sensation sequence, participants did voluntary taps that triggered either auditory beeps or visual flashes as sensory feedbacks. This loop with multiple tap-sensation intervals (with mean interval of 400 or 800 ms) served as a temporal reference for the subsequent comparison of target interval (in a single action-sensation loop). The target interval was defined by a tap with its associated sensory feedback (visual flash or auditory beep). The target interval was 200, 400, 600, 800, 1000, 1200, or 1400 ms for the long mean duration (800 ms) condition and 100, 200, 300, 400, 500, 600, or 700 ms for the short mean duration (400 ms) condition. A typical trial started with a black fixation (“cross” on the monitor screen) which appeared 500 ms before the first signaling tone and lasted until the second cueing tone was over. The first cueing beep (2000 Hz, 500 ms) indicated the start of the action-sensory feedback sequence and prompted the participants to issue the tappings within 3 s. The tap was accompanied with either visual flash or auditory beep, with the repetition of eight action-sensation intervals (mean 400 ms or 800 ms). When the last sensation feedback was over, after a blank interval of 300 ms, participants heard a 2000 Hz beep (200 ms) which indicated the issuing of a last tap for generating target interval (either with visual flash or auditory beep) (Figure 1). We used the method of constant stimuli to compare the target interval duration with the mean action-sensation interval duration. Participants were asked to make a two alternative forced choice (2-AFC) with RTbox, to indicate which interval is longer: the mean action-sensation interval, or the last target interval (Figure 1). We detailed the specific methods for each experiment as follows.

#### Experiment 1

Thirteen participants (with ages from 19 to 25, 6 males) took part in experiment 1. In Experiment 1, we used 500 Hz tones as sensory feedbacks for participants’ voluntary taps. Participants consecutively tapped eight times first, in which each tap was followed by a 500 Hz auditory beep as sensory feedback. The time intervals between action and sensory feedback were not equal (with mean interval of 800 ms and coefficient of variance of 0.1 to 0.15). The target interval was 200, 400, 600, 800, 1000, 1200, or 1400 ms. Participants took two blocks of tests, each block having seven trials for each given target interval. Participants received 14 trials, twice for each target interval, to get familiar with the task.

The data from Experiment 1 served as a subset of baseline data, in which only one type of auditory signals were used. Three further control experiments were implemented to provide baseline data in which only a single type of stimuli was presented eight times, i.e., 500 Hz tones with short intervals, visual flashes with long intervals (mean 800 ms) and visual flashes with short intervals (mean 400 ms). The control experiments were modified after Experiment 1. In addition to the specific mappings of sensory feedbacks and intervals, in each control experiment participants received practices (visual feedback of “correct” or “wrong” after each response) until their accuracies were above 75%. The number of practice blocks were identical to the formal experiments. Thirteen participants (ages from 19 to 24, 5 males) took parts in control experiment (CE1). In CE1 (baseline corresponding to Experiment 2 and Experiment 3), sensory feedbacks were 500 Hz auditory beeps, but the mean tap-beep interval was 400 ms. Thirteen participants (ages from 19 to 24, 3 males) attended in CE2. In CE2 (baseline for Experiment 3), we used visual flashes as sensory feedbacks to associate with the taps. The mean tap-flash interval was 800 ms. Thirteen participants (ages from 18 to 24, 3 males) attended in CE3. In CE3 (baseline for Experiment 3), the tap-visual flash sequence was adopted with the mean tap-flash interval of 400 ms. For all the control experiments, after the preceding sequence was over, the probe interval was given and was always demarcated with the sensory event of the same properties as shown in the sequence. The probe interval was 200, 400, 600, 800, 1000, 1200, or 1400 ms for the long mean duration (800 ms) condition, and 100, 200, 300, 400, 500, 600, or 700 ms for the short mean duration (400 ms) condition.

#### Experiment 2

Seventeen participants (ages from 20 to 25, 5 males) took part in Experiment 2. We used two kinds of auditory feedbacks (500 or 1000 Hz) and two sets of tap-sensation intervals (mean = 400 ms or mean = 800 ms, CVs of both sets of intervals were 0.1 to 0.15). In one condition, short intervals were marked by 500 Hz tones and long intervals were marked by 1000 Hz tones. Nine participants took the test in this condition. In the other condition, eight participants joined the test in which the associations between intervals and tones were reversed (short intervals-high pitch tones and long intervals-low pitch tones). In a tap-sensation sequence, the short and long intervals were mixed. Participants issued eight taps in which the ratio of the short to long intervals was selected from one of the given sets (1:1, 3:5, 5:3). Participants were prompted to compare the target interval with the preceding mean interval of action-sensory feedbacks in four blocks, in which both the target interval and the preceding intervals between action and sensation were marked by the tones with the same pitches. In each block, one target interval (from seven levels) was presented four times. Prior to formal experiment, participants received two tasks for practice. In the first task, they received the practice with both short and long mean intervals (but in one sequence only either 500 or 1000 Hz tones were given). Each target interval was presented three times, resulting in 42 trials. Participants could take another session for practice until their accuracies were above 75%. In the second task, they received another 14 trials (with mixed tones of 500 and 1000 Hz, seven times for each condition). Both practice tasks were implemented with visual feedback of “correct” or “wrong” responses. When the practice session was over, participants took the formal test.

#### Experiment 3

Sixteen participants (ages from 20 to 25, 7 males) took part in Experiment 3. The stimuli configurations and timing parameters were similar to those in Experiment 2, except that the 1000 Hz tones were replaced by visual black disks as sensory feedback. The practice protocol was the same as the one in Experiment 2.

#### Experiment 4

Twelve participants (ages from 20 to 25, 4 males) took part in Experiment 4. The stimuli setting and timing parameters were similar to those in Experiment 2, except that the two sets of action-sensation intervals were same (mean 800 ms) but with different CVs. In one configuration, the intervals marked with 500 Hz tones were associated with CVs of 0.1 to 0.15 (i.e., low variance), and those intervals marked with 1000 Hz were associated with CVs of 0.3 to 0.35 (i.e., high variance). In the other configuration, the mappings between tone pitches and CVs were reversed. Prior to the formal experiment, participants took 14-trial practice with feedback of “correct” or “wrong” responses as did in Experiment 2.

### Data Analysis

In all four experiments, the proportions of reporting the target duration as longer across seven intervals were fitted to the psychometric curve using a logistic function (Treutwein and Strasburger, 1999; Wichmann and Hill, 2001). The transitional threshold, that is, the point of subjective equality (PSE) at which the participant was likely to report the two motion percepts equally, was calculated by estimating 50% of reporting of group motion on the fitted curve. The just noticeable difference (JND), an indicator of the sensitivity of apparent motion discrimination, was calculated as half of the difference between the lower (25%) and upper (75%) bounds of the thresholds from the psychometric curve.

## Results

### Experiment 1 and Control Experiments

#### Exp1

##### Baseline bias when eight sequential stimuli were drawn from a single distribution

**FIGURE 2:**
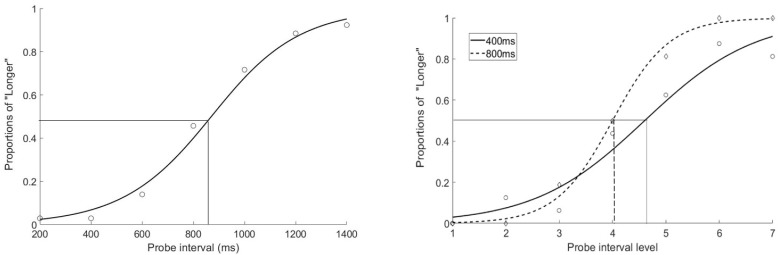
The fitted psychometric curves for Experiment 1 (left, averaged plot) and Experiment 2 (right, plot for a typical participant). The proportions of reporting the probe action-sensation interval as being longer than the mean preceding action-sensation intervals were plotted as a function of the probe intervals (200–1400 ms, with 200 ms as step size, left figure) or probe intervals of different ranges (right figure solid line with short range of 100–700 ms and dashed line with long range of 200–1400 ms). The crossing points on the *x*-axis indicated the PSEs.

**FIGURE 3:**
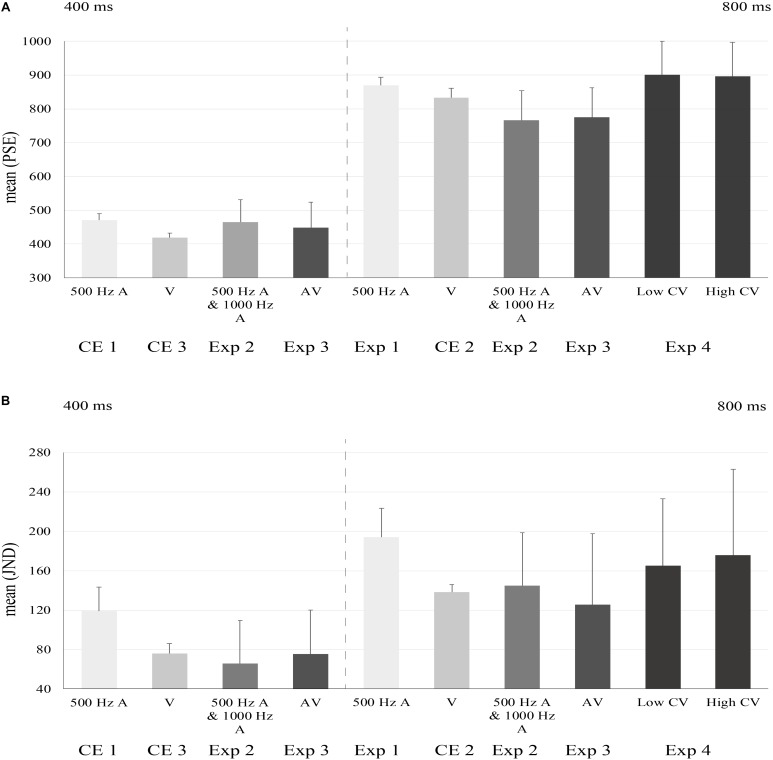
The mean bar plots of PSEs and JNDs for the experiments. A-audio; V-visual;CV-coefficient of variance. The error bar represented the standard errors. The horizontal axis decipted experimental conditions. PSEs and JNDs of Exp2, Exp3, and Exp4 were from data collapsed across tone pitches or modalities of feedbacks. Left halves of **(A)** and **(B)** indicated PSEs and JNDs for short mean duration conditions. Right halves of **(A)** and **(B)** indicated PSEs and JNDs for long mean duration conditions.

The mean PSE and JND were 869.3 ± 24.1 ms (standard deviation) and 194.4 ± 29.4 ms. All the mean PSEs and JNDs were ploted in Figure 3. One sample *t*-test showed that participants underestimated the target interval, compared with 800 ms, *t*(12) = 10.368, *p* < 0.001 (Figure 2, left).

##### Effects of individual standards within the sequence

To evaluate whether certain intervals in the action-sensation sequences play a significant role in determining the estimation of the probe interval, e.g., the potential recency effect stemming from the last interval (Wan and Chen, 2018), we performed binary logistic regression with responses to target intervals (“0” as shorter and “1” as longer compared with the mean interval) as dependent variable and eight sequential intervals and probe interval as predictor variables for each participant. Ominibus Tests of Model Coefficients of all participants’ model reached significant level (*p*s < 0.001), which suggested at least one of the predictor variables was statistically significant in contributing the discrimination of probe interval. The results of Hosmer and Lemeshow Tests of models were not significant (*p*s > 0.143), implying good fitness of the models. We then implemented one-sample *t*-tests comparing parameter estimates of the eight sequential intervals of all participants with “0.” None of these sequential intervals reached significant level (*p*s > 0.521). Finally, a repeated-measure ANOVA test was implemented with positions of sequential intervals as within-subject variables on parameter estimates of sequential intervals of all participants. The difference between sequential intervals was partially significant [*F*(7,84) = 2.112, *p* = 0.051, *η*^2^ = 0.150] and the effect of intercept was not significant [*F*(1,12) = 0.291, *p* = 0.599, *η*^2^ = 0.024]. The detailed values were given in Table 1.

**Table 1:** The parameter estimates of binary logistical regressions. The probe intervals were labeled as 1∼7 in the regression models.

	Temporal averaging								
	interval 1	interval 2	interval 3	interval 4	interval 5	interval 6	interval 7	interval 8	constant
Exp 1	10.339 (0.612)	13.537 (0.521)	11.972 (0.548)	12.492 (0.551)	10.027 (0.628)	7.445 (0.688)	11.265 (0.563)	7.520 (0.710)	-71.002 (0.584)
CE 1	34.634 (0.055)	35.504 (0.063)	34.510 (0.069)	34.092 (0.096)	29.171 (0.143)	30.904 (0.125)	34.241 (0.079)	34.853 (0.085)	-113.509 (0.074)
CE 2	17.221 (0.338)	15.470 (0.431)	15.116 (0.417)	14.143 (0.467)	17.329(0.364)	14.501 (0.453)	15.829 (0.384)	11.688 (0.539)	-100.490 (0.410)
CE 3	29.956 (3.15)	23.346 (0.418)	27.562 (0.348)	28.102 (0.346)	30.534(0.309)	28.021 (0.310)	26.814 (0.358)	28.823 (0.277)	-92.127 (0.319)
Exp 2	-0.335 (0.295)	-0.611 (0.135)	-0.518 (0.067)	-0.608 (0.081)	-0.472 (0.123)	-0.952 (0.010)**	-0.952 (0.010)**	1.070 (0.006)	-1.027 (0.424)
Exp 3	-0.318 (0.620)	-0.478 (0.299)	-0.464 (0.385)	-0.774 (0.165)	-0.515(0.259)	-1.105 (0.042)*	-0.494 (0.077)	-0.2824 (0.624)	-1.557 (0.371)
Exp 4	-6.482 (0.110)	-7.008 (0.085)	-5.687 (0.112)	-7.191 (0.093)	-6.324(0.130)	-6.260 (0.115)	-6.264 (0.118)	-6.369 (0.113)	57.965 (0.140)


*The values in the table indicated the corresponding mean beta values in the regression models. The values in the brackets referred to the *p*-values corresponding the beta values in one sample *t*-test (^∗^*p* < 0.05; ^∗∗^*p* < 0.01).*

#### CE1

In this separate control experiment with 500 Hz auditory beeps and short mean durations, the mean PSE and JND were 470.8 ± 19.5 ms and 119.1 ± 24.5 ms. One sample *t*-test revealed a significant bias of perceived “compression” of the probe intervals (compared with the reference of 400 ms) [*t*(12) = 13.333, *p* < 0.001]. Binary logistic regression, the same as in Exp1 was applied. Ominibus Tests of Model Coefficients of all models reached significant level (*p*s < 0.001). The results of Hosmer and Lemeshow Tests of models were not significant (*p*s > 0.196) for eleven participants except for two participants (which means their models were not good fitted). Thus we implemented one-sample *t*-tests with the two participants excluded. None of these sequential intervals reached significant level (*p*s > 0.055). The repeated measures ANOVA test revealed a partially significant effect of intercept [*F*(1,12) = 4.585, *p* = 0.053, *η*^2^ = 0.276] but no significant effect of sequential intervals [*F*(7,84) = 0.702, *p* = 0.610, *η*^2^ = 0.055].

#### CE2

The mean PSE and JND of the control experiment with visual flashes and long mean duration (800 ms) were 832.7 ± 27.6 and 138.2 ± 7.5 ms. One sample *t*-test of this condition showed participants’ tendency of “compressing” probe intervals as above [*t*(12) = 4.271, *p* = 0.001] (Figure 3). Ominibus Tests of Model reached significant level (*p*s < 0.001) and Hosmer and Lemeshow Tests of models were not significant [*p*s > 0.579] for the binary logistic regression. One-sample *t*-tests showed that none of the effects of these sequential intervals were significant (*p*s > 0.345). Both the effects of sequential intervals [*F*(1.000,12.003) = 1.007, *p* = 0.335, *η*^2^ = 0.077] and intercept [*F*(1,12) = 0.958, *p* = 0.347, *η*^2^ = 0.074] were not significant by repeated-measure ANOVA test.

#### CE3

For the control experiment with visual flashes and short mean duration (400 ms), the mean PSE and JND were 418.5 ± 13.0 and 75.7 ± 10.3 ms. Participants had biases to “compress” the probe intervals [*t*(12) = 5.128, *p* < 0.001]. For binary logistic regressions, Ominibus Tests of Model reached significant (*p*s < 0.001) and Hosmer and Lemeshow Tests of models were not significant (*p*s > 0.364). One-sample *t*-tests showed none of these sequential intervals was significant in contributing the perceived probe intervals (*p*s > 0.277). The repeated-measure ANOVA test showed neither effect of sequential intervals [*F*(1.001,12.015) = 1.018, *p* = 0.333, *η*^2^ = 0.078], nor effect of intercept [*F*(1,12) = 0.960, *p* = 0.347, *η*^2^ = 0.074].

##### Combine data from Exp1 and CEs for analysis

A 2 × 2 ANOVA test that took modality (auditory/visual) and mean duration (short/long) as between-subject factors showed, for both PSEs and JNDs, a significant main effect of modality [PSE: *F*(1,48) = 54.890, *p* < 0.001, *η*^2^ = 0.533; JND: *F*(1,48) = 79.144, *p* < 0.001, *η*^2^ = 0.622] and a significant main effect of mean duration [PSE: *F*(1,48) = 4577.967, *p* < 0.001, *η*^2^ = 0.990; JND: *F*(1,48) = 151.808, *p* < 0.001, *η*^2^ = 0.760]. The interaction of modality and mean duration was not significant [PSE: *F*(1,48) = 1.725, *p* = 0.195, *η*^2^ = 0.035; JND: *F*(1,48) = 1.314, *p* = 0.257, *η*^2^ = 0.027]. To be more specific, PSEs and JNDs of auditory modality were significantly larger than those in visual modality. PSEs and JNDs in short mean duration condition was significantly smaller than those in long mean duration condition.

The data from Exp1 and CEs could serve as control references for following experiments.

### Experiment 2

#### Sequential Stimuli With Two Different Interval Distributions Around Two Alternative References (Standards)

The mean PSE and mean JND of probe intervals for “500 Hz–400 ms” condition in “1000 Hz–800 ms” context were 440.0 ± 58.3 and 84.8 ± 33.7 ms. The mean PSE and mean JND of “1000 Hz–400 ms” in “500 Hz–800 ms” context were 493.0 ± 65.8 and 120.3 ± 47.8 ms (Figure 2, right). The mean PSE and mean JND of “1000 Hz–800 ms” in “500 Hz–400 ms” context were 750.8 ± 96.2 and 146.1 ± 59.9 ms. The mean PSE and mean JND of “500 Hz–800 ms” in “1000 Hz–400 ms” context were 784.5 ± 77.0 and 143.8 ± 49.5 ms.

We performed a repeated measures analysis of variance (ANOVA) test with context from different matchings between tones (500 Hz, 1000 Hz) and means of intervals (400 ms, 800 ms) as between-subject variable, and means of sequential intervals as within-subject variable. There was no significant main effect of context [*F*(1,15) = 2.795, *p* = 0.115, *η*^2^ = 0.157] but interval means had a significant main effect on PSEs [*F*(1,15) = 131.618, *p* < 0.001, *η*^2^ = 0.898]. For JNDs, context also did not make a difference [*F*(1,15) = 0.740, *p* = 0.403, *η*^2^ = 0.047]. However, the main effect of the mean interval duration was significant [*F*(1,15) = 9.704, *p* = 0.007, *η*^2^ = 0.393]. This result pattern indicated that participants had selectively extracted different “mean” intervals to make prompt perceptual decision of the probe intervals. Therefore, we collapsed the data across two types of tone pitches for further analysis. The mean PSEs for short and long mean durations (across both pitches) were 464.9 ± 65.9 and 766.7 ± 86.7 ms. The mean JNDs for short and long mean durations were 101.5 ± 43.6 and 145.0 ± 53.6 ms.

#### Comparison Between Exp2 and Corresponding Control Experiments for Short and Long Mean Conditions

We implemented one-way ANOVA to compare the collapsed data and data from corresponding control experiments, i.e., Experiment 2 and CE1. For short mean duration condition, we did one-way ANOVA with context as between-subject variable. The context included three conditons: 500 Hz with short mean duration control (i.e., “500 Hz–400 ms”), 500 Hz with short mean duration stimuli in the context of 1000 Hz long mean duration stimuli (“500 Hz–400 ms and 1000 Hz–800 ms”), 1000 Hz with short mean duration stimuli in the context of 500 Hz long mean duration stimuli (“500 Hz–800 ms and 1000 Hz–400 ms”). The effect of context was not significant on PSEs [*F*(2,27) = 2.650, *p* = 0.089]. The context also didn’t make a difference on JNDs [*F*(2,27) = 3.190, *p* = 0.057].

For long mean duration condition, the same one-way ANOVA test was implemented. The results showed that the context had a significant effect on PSEs [*F*(2,27) = 9.072, *p* = 0.001]. PSEs of “500Hz–800 ms” control was significantly larger than both PSEs of “500 Hz–800 ms” in “1000 Hz–400 ms” context (*p* = 0.015) and PSEs of “1000 Hz–800 ms” in “500 Hz–400 ms” context (*p* = 0.009). Also, there was a significant main effect of context on JNDs [*F*(2,27) = 4.307, *p* = 0.024]. However, JND of “500 Hz–800 ms” in “1000 Hz–400 ms” context were marginally significantly different from JND of “500 Hz–800 ms” control [*p* = 0.061]. JND of “1000 Hz–800 ms” in “500 Hz–400 ms” context was the same as the JND of “500 Hz–800 ms” control (*p* = 0.110).

#### Effects of Individual Standards Within the Sequence

Binary logistic regressions analysis was applied to Experiment 2 as in Experiment 1. For all participants, results of Ominibus Tests of Model Coefficients reached significant level (*p*s < 0.001) and results of Hosmer and Lemeshow Tests of models were not significant (*p*s > 0.250). One-sample *t*-tests comparing parameter estimates of 8 sequential intervlas with 0 revealed that the last three sequential intervals contributed to participants’ responses (*p*s < 0.010). A repeated measures ANOVA test was done as in Exp1. There was no significant effect of sequential intervals [*F*(7,1112) = 0.898, *p* = 0.511, *η*^2^ = 0.053] but the effect of intercept was significant [*F*(1,16) = 13.675, *p* = 0.002, *η*^2^ = 0.461]. This result pattern indicated that with two standards of references (sequences), participants could have some initial preferences responding to the specific sequence (short vs. long). Moreover, with the increasing complexity of stimuli, participants depended more on the recent intervals to make perceptual decision for the probe interval.

Therefore, with mixed and complicated action-sensation sequences, observers could extract selectively the mean intervals of specific action-sensation sequence to facilitate the temporal discriminations for the probe intervals. However, due to the to the repetition effect with the multiple intervals (Pariyadath and Eagleman, 2007; Matthews and Meck, 2014; Matthews and Gheorghiu, 2016), the perceived mean interval has been shortened compared with one standard (long) mean interval with the single sequence. This “compression” effect has attracted and biased the probe interval to be subjectively perceived as shorter (with larger PSEs). We’ll come to this point in the Discussion section.

### Experiment 3

#### Sequential Stimuli With Two Different (Auditory and Visual) Interval Distributions Around Two Alternative References (Standards)

The mean PSE and mean JND of “A(uditory) – 400 ms” in “V(isual) – 800 ms” context were 456.2 ± 64.2 and 86.9 ± 47.8 ms. The mean PSE and mean JND of “V – 400 ms in A – 800 ms” context were 439.5 ± 88.5 and 104.0 ± 42.7 ms. The mean PSE and mean JND of “V – 800 ms” in “A – 400 ms” context were 784.3 ± 108.3 and 117.1 ± 77.6 ms. The mean PSE and mean JND of “A – 800 ms” in “V – 400ms” context were 764.3 ± 68.0 and 133.9 ± 70.9 ms (Figure 3). A repeated measures ANOVA analysis with mean of action-sensation intervals (400 or 800 ms) as within-subject variable and context of different mappings between stimuli (visual flashes and auditory beeps) with the short/long intervals, indicated there were no significant influence of context [PSE: *F*(1,14) = 0.414, *p* = 0.530, *η*^2^ = 0.029; JND: *F*(1,14) = 0.360, *p* = 0.558, *η*^2^ = 0.025]. However, the main effect of the mean intervals was significant on PSEs [*F*(1,14) = 111.644, *p* < 0.001, *η*^2^ = 0.889] and JNDs [*F*(1,14) = 6.229, *p* = 0.026, *η*^2^ = 0.308]. Therefore, we collapsed the data across stimuli types (auditory vs. visual). The mean PSEs for short and long mean interval conditions were 447.8 ± 75.2 and 774.3 ± 88.0 ms. The mean JNDs for short and long mean interval conditions were 95.4 ± 44.7 and 125.5 ± 72.3 ms (Figure 3).

#### Comparison Between Exp3 and Corresponding Control Experiments for Short and Long Mean Conditions

As above, we implemented a two-way ANOVA test on the collapsed data and corresponding control data for short mean duration condition, with modality of feedbacks (auditory beeps/visual flashes) and context (context of 500 Hz–400 ms control/context of Exp 3) as between-subject variables. For PSEs, there was no significant interaction effect of modality × context [*F*(1,38) = 2.434, *p* = 0.127, *η*^2^ = 0.060]. The modality of sensory feedbacks had a significant effect [*F*(1,38) = 6.686, *p* = 0.014, *η*^2^ = 0.150] but the context didn’t have such a significant effect [*F*(1,38) = 0.034, *p* = 0.855, *η*^2^ = 0.001]. The PSEs of “A – 400 ms” in “V – 800 ms” context in Exp3 were not different from PSEs of “A – 400 ms” in CE1 (*p* = 0.225). The PSEs of “V – 400 ms” in “A – 800 ms” context were the same as PSEs of “V – 400 ms” in CE3 [*p* = 0.336]. For JNDs, there was a significant effect of modality × context [*F*(1,38) = 14.152, *p* = 0.001, *η*^2^ = 0.271]. The results also revealed a significant effect of modality [*F*(1,38) = 5.458, *p* = 0.025, *η*^2^ = 0.126] but not of context [*F*(1,38) = 0.576, *p* = 0.452, *η*^2^ = 0.015]. The JNDs of “A – 400 ms” in “V – 800 ms” context in Exp3 were smaller than JNDs of “A – 400 ms” in CE1 (*p* = 0.003) and the JNDs of “V – 400 ms” in “A – 800 ms”in Exp3 were, however, larger than JNDs of “V – 400 ms” in CE3 (*p* = 0.040).

For long mean duration coditon, the same two-way ANOVA test was implemented. We didn’t find significant interaction effect of modality × context on PSEs [*F*(1,38) = 0.542, *p* = 0.466, *η*^2^ = 0.014]. The modality made no difference for PSEs [*F*(1,38) = 2.205, *p* = 0.146, *η*^2^ = 0.055]. But context had a significant effect on PSEs [*F*(1,38) = 9.741, *p* = 0.003, *η*^2^ = 0.204]. The PSEs of “A – 800 ms” in “V – 400 ms” context in Exp3 were significantly larger than PSEs of “A – 800 ms” context in Exp1 (*p* = 0.010) The PSEs of “V – 800 ms” in “A – 400 ms” context in Exp3 were the same as PSEs of “V – 800 ms” context in CE2 (*p* = 0.100). For JNDs, only modality had a significant effect [*F*(1,38) = 8.732, *p* = 0.005, *η*^2^ = 0.187]. There was no significant interaction of modality × context [*F*(1,38) = 0.238, *p* = 0.628, *η*^2^ = 0.006] or effect of context [*F*(1,38) = 3.186, *p* = 0.082, *η*^2^ = 0.077]. There were no differences between JNDs of “A – 800 ms” in “V – 400 ms” context in Exp3 and of “A – 800 ms” context in Exp1 (*p* = 0.116) or between JNDs of “V – 800 ms” in “A – 400 ms” context in Exp3 and the JNDs of “V – 800 ms” context in CE2 (*p* = 0.365).

#### Effects of Individual Standards Within the Sequence

The binary logistic regressions showed good fit for 15 participants: Ominibus Tests of Model Coefficients reached significant level (*p*s < 0.001) but Hosmer and Lemeshow Tests of models were not significant (*p*s > 0.163). The result showed that seven of eight sequential intervals alone could not predict participants responses [*p*s > 0.066] but the sixth one contributed to participants’ reponses (*p* = 0.042). The results of repeated-measure ANOVA test showed no effect of sequential intervals [*F*(3.995,59.919) = 0.335, *p* = 0.853, *η*^2^ = 0.022] but a significant effect of intercept [*F*(1,15) = 5.204, *p* = 0.038, *η*^2^ = 0.258].

### Experiment 4

#### Sequential Stimuli With Two Different Variances but With the Same Mean Reference Duration

We implemented a two-way repeated measures ANOVA test to examine whether various mappings of tone pitches (500 Hz vs. 1000 Hz) and CVs (0.1–0.15 vs. 0.3–0.35) made a difference. The results indicated that orthogonal mappings did not make a difference [*F*(1,10) = 0.988, *p* = 0.344, *η*^2^ = 0.090]. Therefore, we collapsed the data across tone piches as did in Exp2. The mean PSEs for low CV and high CV interval conditions were 900.4 ± 99.1 and 895.8 ± 101.6 ms, and the mean JNDs under the two CVs were 165.0 ± 68.1 and 175.6 ± 87.9 ms.

#### Comparison Between Exp 4 and Corresponding Control Experiments

One-way ANOVA test with CV (low/high/control) indicated that there was no significant main effect either on PSEs [*F*(2,34) = 0.533, *p* = 0.591] or on JNDs [*F*(2,34) = 0.645, *p* = 0.531]. Again, binary logistic regressions for all participants showed that Ominibus Tests of Model reached significant level (*p*s < 0.001) and Hosmer and Lemeshow Tests of models were not significant (*p*s > 0.138). One-sample tests suggested none of these sequential intervals were significant (*p*s > 0.093). Finally, a repeated- measure ANOVA test was implemented. No differences between sequential intervals were found [*F*(2.389,26.278) = 0.509, *p* = 0.639, *η*^2^ = 0.044] and the effect of intercept was not significant [*F*(1,11) = 3.124, *p* = 0.105, *η*^2^ = 0.221].

## Discussion

In current study we reported that humans are able to use the mean of multiple irregular action-sensation intervals, to compare with the subsequent probe interval which was defined by a single tap and its sensation (visual flash or auditory beep). However, during this comparison, human observers might use only some of the intervals rather than all of them.

This temporal averaging ability has been robustly observed in the loop of action-sensation (sensory feedback) as did in the pure perceptual domian (with a sequence of stimuli) (Jazayeri and Shadlen, 2010; Shi et al., 2013; Karaminis et al., 2016; Wan and Chen, 2018). Importantly, human observers can selectively average the mean of the multiple intervals between action and sensations. This selectivity was demonstrated in two aspects: (1) Tuning to short and long intervals. In current configurations, we implemented short mean interval (400 ms) and long mean interval (800 ms) conditions by presenting a sequence containing the voluntary actions and their associated auditory beeps as sensory feedback (Experiments 1, 2, and 4). Participants could adaptively make the discrimination of the probe interval and referred to either the “short” standard or “long” standard (mean) intervals being extracted. (2) Selectivity across different sensory modalities. In Experiment 3, we mixed the auditory beeps and visual flashes in the same action-sensation loop. Participants could judge the probe interval by picking up the corresponding specific sequence, summarized mean tap-tone interval or tap-flash interval to facilitate the discrimination of the probe interval (either “auditory” or “visual” event as the final marker in the probe). Temporal averaging of time intervals between action and sensation is relatively robust. The ability to average the mean intervals were less influenced by the distribution profile (as shown in the low vs. high variances) of the intervals Human observers calculate different temporal ranges (short vs. long), irrespective of the intersensory bindings of the differential temporal ranges or different sensory events (Chen and Vroomen, 2013), or with different variabilities of the intervals themselves (Acerbi et al., 2012).

This robust temporal averaging between action and sensation was achieved by a similar mechanism of central tendency effect (Jazayeri and Shadlen, 2010; Burr et al., 2013; Shi et al., 2013; De Corte and Matell, 2016a; Karaminis et al., 2016), in which the perceptual discrimination of the probe/target inteval was biased to the mean interval of the preceding mean action-sensation intervals.

As shown in the literature of timing research, perception of temporal synchrony/asynchrony between one’s own action and the sensory feedback of that action is quite flexible, in which the time order of cause (action) and effect (sensory feedback) could even be reversed due to the repetitious adaptation (Stetson et al., 2006; Heron et al., 2009; Sugano et al., 2010, 2012, 2014; Acerbi et al., 2012; Keetels and Vroomen, 2012). This flexibility has been shown in different forms. Human observers could simultaneously adapt to differential intersensory temporal bindings in audiovisual speech (Overduin et al., 2008; Heron et al., 2009, 2012; Roseboom and Arnold, 2011; Curran et al., 2012; Yuan et al., 2012; McWalter and McDermott, 2018) and in (hands) action-sensation couplings (Sugano et al., 2014). For the audiovisual temporal recalibration effect, humans can form multiple simultaneous estimates of differential timing for audiovisual synchrony, in which the positive or negative temporal asynchronies between auditory and visual streams (identified by associating with either the male or female speech) led to the corresponding shifts of temporal relations, after “selective” adaptations to one of the two temporal relations (Roseboom and Arnold, 2011). This concurrent recalibration effect has been demonstrated in a clever design in which Sugano et al. (2014) exposed the participants’ left and right hands to different action-sensory feedback lags (“clicks”), one for long delay (∼150 ms) and one for short delay subjective no-delay (∼50 ms). In addition to observing the traditional temporal recalibration effect, Sugano et al. (2014) found different effectsizes of TRs due to the differential “delayed” feedbacks. Those findings indicated that human observers have both central and motor/sensory specific timing processing mechanisms in dealing with the temporal bindings between events and actions (Chen and Vroomen, 2013; Ivry and Schlerf, 2008).

In the current study, though the central tendency effect was robustly replicated in the sensorimotor domain, we did not observe a fixed pattern of the potential recency effect, i.e., the potent role of the last interval in action-sensation sequence (Burr et al., 2013). And interestingly, we did not find a distinctive change in the behavioral performance with respective to the modalities (auditory vs. visual sensory events). This finding is largely against the established knowledge of auditory dominance (with high temporal precision) over visual signal in sensory timing and in sensorimotor recalibration (Burr et al., 2009; Lukas et al., 2014; Sugano et al., 2016). However, one typical finding is that the perceived probe intervals were longer in long mean auditory intervals context (“A – 800 ms” in “V – 400 ms”) compared with the ones in “A – 800 ms” (baseline), but no bias for the long mean visual intervals counterpart. This pattern indicates that we still keep the sensitivity for more salient and accurately timing stimuli–auditory beeps and are hence subject to the contextual modulation.

Using the mean intervals in action-sensation loop to compare with the subsequent probe interval could be attentional resource- consuming, which constrains the otherwise “advantage” of auditory events (Cheng et al., 2014). During the unfolding of the action-sensation loop, participants should always hold in the working memory of the many intervals (Van Rijn, 2016), and switch frequently of intervals with different durations and with different sensory events (visual flashes and auditory beeps). In this context, we suggest that the fine distinction of the last interval has been interfered and concealed to impose the potentially observable influence on discriminating the probe action-sensation interval. Nevertheless, to maintain and exploit the grossly “abstract” means is less demanding and is even automatically acquired, as shown in a large body of literature (Chong and Treisman, 2003; Haberman et al., 2009; Haberman and Whitney, 2009; de Gardelle and Summerfield, 2011; Albrecht et al., 2012; Piazza et al., 2013). In our case, with the unfolding of the action-sensation sequence, we had to hold in the (working) memory with multiple intervals and multiple sensory events before we made perceptual decision of the probe interval. This increased number of items in memory, as well as the interference of holding two standards (short vs. long mean intervals), and time decay between the preceding sequence and the probe, could be challenging to one’s limited capacity of information processing (Cheng et al., 1996, 2014). However, we did not observe this detriment in present tasks. Note that the total time span for all the events in a sequence was about 7 s, which was shorter than the pure time-delay (last above 30 s) between the offset of the sequence (stimuli) and the probe stimuli in other relevent studies (Jones and Wearden, 2004; Ogden et al., 2008), where the long delay is subject to the memory decay (interference). Therefore, in our case, we believe participants could well maintain the events in memory and mobilize the attentional resources to fullfil the tasks.

The control experiments with only one standard (mean duration of 400 or 800 ms), with the comparsion of the corresponding main exepriments, further supported that a robust averaging has been observed, even though there were general biases in which the perceived (mean) time interval was “compressed” with mixed sequences (“standards”) and had been observed obviously with “short” standard. This illusory “compression” of perceived time interval could be elicited by the repetition effect of extended, complex structures of events, which lead to the subjectively “shortened” element interval (Sasaki et al., 2002; Nakajima et al., 2004; Matthews and Meck, 2014, 2016; Matthews and Gheorghiu, 2016). Alternatively, the direct attention on the multiple stimuli (or distraction on the stimuli) that demarcating the intervals, would somehow consume the resources for processing the “intervals” themselves (hence the less attended intervals were preceived as shorter) which could lead to the observed “compression” effect (Mattes and Ulrich, 1998; Tse et al., 2004). The direct attention across auditory or visual events, and the attentional switching between different sensory events, also contributed to the imbalance of perceiving the same physical intervals. For example, in the control test, the mean 800 ms in tap-beep sequence was indeed perceived as shorter than the 800 ms in the tap-visual flash sequence. It is probably due to the expansion of intervals by the onset of visual events, especially when the visual events were dyanmic and unexpected (Kanai et al., 2006; Kanai and Watanabe, 2006).

With that said, we should pay attention to the limitations of current studies. For instance, we did not test empirically how the efficiency of using the mean intervals in sensorimotor domain is constrained by the invidiviual working memory capacity. We are also not informed how the degrees of complexity of the temporal structure (including the more levels of CVs for the durations) would affect the “averaging” processing. Further research evidence is needed to address these considerations.

In sum, we revealed a novel and robust temporal averaging process in sensorimotor domain, by employing the action-sensory intervals as building elements in the perception-action sequence. Our findings suggest that human observers can use the mean action-sensation intervals to facilitate and optimize the task-relevant perceptual decision for the subsequent time information in the critial action- sensation loop. The robust averaging of action-sensation intervals suggests that a centralized timing mechanism may subserve this process (Ivry and Schlerf, 2008), though it is constrained and even interfered by contextual factors (Jazayeri and Shadlen, 2010; Cheng et al., 2014; De Corte and Matell, 2016b), including memory mixing (Van Rijn, 2016) and attentional-capacity limitations (Cheng et al., 2014) and some contributions of salient individual events in the loop.

## Author Contributions

LC designed the study. HZ and LC analyzed the data and wrote the manuscript.

## Conflict of Interest Statement

The authors declare that the research was conducted in the absence of any commercial or financial relationships that could be construed as a potential conflict of interest.
